# Using the robust principal component analysis algorithm to remove RF spike artifacts from MR images

**DOI:** 10.1002/mrm.25851

**Published:** 2015-07-20

**Authors:** Adrienne E. Campbell‐Washburn, David Atkinson, Zoltan Nagy, Rachel W. Chan, Oliver Josephs, Mark F. Lythgoe, Roger J. Ordidge, David L. Thomas

**Affiliations:** ^1^ Centre for Advanced Biomedical Imaging, University College LondonLondonUnited Kingdom; ^2^ Division of Intramural ResearchCardiovascular and Pulmonary BranchNational Heart, Lung, and Blood Institute, National Institutes of HealthBethesdaMarylandUSA; ^3^ Centre for Medical Imaging, University College LondonUnited Kingdom; ^4^ Wellcome Trust Centre for Neuroimaging, UCL Institute of Neurology, University College LondonLondonUnited Kingdom; ^5^ Laboratory for Social and Neural Systems Research (SNS Lab)Department of EconomicsUniversity of ZurichZurichSwitzerland; ^6^ Birkbeck–UCL Centre for Neuroimaging, Birkbeck CollegeLondonUnited Kingdom; ^7^ Department of Anatomy and NeuroscienceUniversity of MelbourneMelbourneAustralia; ^8^ Department of Brain Repair and RehabilitationUCL Institute of Neurology, University College LondonLondonUnited Kingdom

**Keywords:** spike noise, robust principal component analysis, stripe artifact

## Abstract

Brief bursts of RF noise during MR data acquisition (“k‐space spikes”) cause disruptive image artifacts, manifesting as stripes overlaid on the image. RF noise is often related to hardware problems, including vibrations during gradient‐heavy sequences, such as diffusion‐weighted imaging. In this study, we present an application of the Robust Principal Component Analysis (RPCA) algorithm to remove spike noise from k‐space. **Methods**: Corrupted k‐space matrices were decomposed into their low‐rank and sparse components using the RPCA algorithm, such that spikes were contained within the sparse component and artifact‐free k‐space data remained in the low‐rank component. Automated center refilling was applied to keep the peaked central cluster of k‐space from misclassification in the sparse component. **Results**: This algorithm was demonstrated to effectively remove k‐space spikes from four data types under conditions generating spikes: (i) mouse heart T_1_ mapping, (ii) mouse heart cine imaging, (iii) human kidney diffusion tensor imaging (DTI) data, and (iv) human brain DTI data. Myocardial T_1_ values changed by 86.1 ± 171 ms following despiking, and fractional anisotropy values were recovered following despiking of DTI data. **Conclusion**: The RPCA despiking algorithm will be a valuable postprocessing method for retrospectively removing stripe artifacts without affecting the underlying signal of interest. Magn Reson Med 75:2517–2525, 2016. © 2015 The Authors. Magnetic Resonance in Medicine published by Wiley Periodicals, Inc. on behalf of International Society for Magnetic Resonance in Medicine. This is an open access article under the terms of the Creative Commons Attribution License, which permits use, distribution and reproduction in any medium, provided the original work is properly cited.

## INTRODUCTION

Artifacts in MR images degrade image quality and can affect the quantitative values derived from the images. Stripe artifacts are common and are caused by radiofrequency (RF) noise from a variety of sources including hardware problems of the MR system, such as vibrations during gradient‐heavy sequences, loose connections in RF or gradient coils, or improper shielding, as well as factors such as the subject's clothing, low humidity, faulty electrical insulation. Short bursts of random RF noise create high intensity spikes in k‐space, resulting in stripes across the image, the exact appearance of which depends on the location of the spike in k‐space. This artifact is particularly prominent in gradient‐heavy sequences such as diffusion tensor imaging (DTI) 1. Ideally, imperfections in hardware should be addressed promptly; however, the source of the RF spikes is notoriously difficult to find, and thus in practice, data is sometimes acquired when these faults are present and the resulting artifacts must be removed in image postprocessing. Previous postprocessing algorithms have been developed to detect spikes in k‐space based on user‐defined thresholds 2, by comparison with a series of similar images 1, 3, or by Fourier transforms of the background noise signal 4, 5. We sought to develop an alternative method that is semiautomated and can reliably remove image artifacts for a broad range of data types.

The work described in this study is based on the principle that k‐space spikes can be isolated using a decomposition of the k‐space matrix into its sparse and low‐rank components. Several frameworks for this decomposition have emerged recently 6, 7, 8, and have been used for video surveillance and facial recognition. In addition, such decompositions have been used for applications within MRI, such as separation of dynamic and static components of an image series for motion and contrast enhancement 9, 10.

Here, we present an algorithm that applies Robust Principal Component Analysis (RPCA) 6 to remove RF spike noise from k‐space. The k‐space matrix is decomposed, such that the RF spikes are in the sparse component and the artifact‐free k‐space is in the low‐rank component. We demonstrate the versatility of this algorithm by applying it to four different data types acquired during periods of RF spiking: T_1_ mapping in the mouse heart, cine imaging of the mouse heart, DTI data in the human kidney and DTI data in the human brain. Application of the despiking algorithm improves image quality and recovers quantitative parameter values derived from the images.

## METHODS

### Despiking Algorithm

We used two steps to remove RF spikes from k‐space: (i) apply RPCA to identify spikes in corrupted k‐space data and (ii) refill pixels at the peaked center of k‐space incorrectly classified as sparse, as follows:

(i) RPCA aims to decompose a measured matrix (**M**) into a low‐rank matrix (**L**) and a sparse matrix (**S**), by solving the optimization problem: $\min_{L,S}{\left\|L\right\|}_{*}+\lambda {\left\|S\right\|}_{1}$ subject to **M** = **L**+**S**_,_ where ${\left\|\cdot \right\|}_{*}$ represents the nuclear norm of a matrix and ${\left\|\cdot \right\|}_{1}$ represents the L1‐norm of a matrix. Conventional PCA typically seeks the best low rank representation of data, in a least square sense, using a small number of principal components. The number of principal components, chosen by the user, determines the rank. Conventional PCA can be applied to a data covariance matrix or directly to the raw data (typically using a singular value decomposition algorithm). The “Robust PCA” algorithm operates directly on the raw data to find a low‐rank estimate of the data that is robust to arbitrarily large outliers 6. The user does not specify the rank of **L**, and data that does not fit a low‐rank representation is contained within an additional term—the sparse matrix—which can have arbitrarily large values.

In the case of RF spike noise, **M** represents the measured data, **S** represents the high intensity RF spikes, and **L** represents the recovered artifact‐free k‐space data. For multiframe data, **M** is arranged as a k‐t matrix (i.e., each full k‐space is a column in the matrix), and the ordering of the frames within this Casorati matrix **M** has no impact on the RPCA decomposition. In a series of images, the sparse component contains the frame‐to‐frame changes that are not explained by the low‐rank component. When analyzing only a single image frame, **M** is k_x_‐k_y_ matrix and the sparse component contains the line‐to‐line data not explained by the low‐rank component.

The default value of λ was $\frac{1}{\sqrt{{(N}_{v}\cdot N_{t})}}$ where N_v_ is the image matrix size (N_v_ = N_x_⋅N_y_) and N_t_ is the number of frames 6. Because k‐space is highly peaked near the center, we multiplied the default value of λ by a factor κ, that increases the sparsity penalty in the cost function 6. In this case, the optimization problem becomes ${\text{min}}_{L,S}{\left\|\left\|L\right\|\right\|}_{*}+\;\lambda_{\text{eff}}{\left\|\left\|S\right\|\right\|}_{1}$, where $\lambda_{\text{eff}}=\;\kappa \cdot \lambda$ For each data type, a range of κ values were tested and the resulting decompositions (**L** and **S**) were compared visually to choose an optimal κ value. If κ is set too low, a larger region at the center of k‐space is included in the sparse component. If κ is set too high, the center is correctly assigned to the low‐rank component, but the spikes are not fully eliminated from the low‐rank component. κ was chosen in a single dataset, and the same value was applied to all other datasets of the same type.

RPCA was performed in MATLAB R2013a (Mathworks, Natick, MA) using the Augmented Lagrange Multiplier (ALM) method, “inexact_alm_rpca.m” (http://perception.csl.illinois.edu/matrix‐rank/sample_code.html), based on the algorithm presented by Lin et al 11. We modified the “inexact_alm_rpca.m” algorithm to accept complex k‐space data.

(ii) To undo any misclassification of the peaked central region of k‐space as sparse, we automatically refilled the pixels at the central cluster of k‐space from the sparse matrix to the low‐rank matrix. Non‐zero values in **S** in the central 16 × 16 pixels, and all connected pixels (using “bwlabel.m” with default 8‐connectivity), were refilled in **L**. The low‐rank matrix, **L**, was then Inverse Fourier Transformed to create the despiked corrected images.

### Mouse Heart T_1_ Mapping

Animal work was undertaken in accordance with the UK Animals (Scientific Procedures) Act 1986 and local ethical guidelines. T_1_ mapping in the mouse heart was performed at 9.4 Tesla (T) (Agilent Technologies, Santa Clara, CA) using a segmented ECG‐gated Look‐Locker method 12 (echo time/repetition time [TE/TR] = 1.18 ms/3 ms, flip angle = 5°, resolution = 200 μm, slice thickness = 1.5 mm, matrix = 128 × 128, recovery delay = 8 s, 50 images). The despiking algorithm was applied to 12 spike‐corrupted datasets acquired using a malfunctioning volume resonator coil. Artifact reduction was visually assessed following despiking and quantitative T_1_ values were compared. T_1_ was estimated in MATLAB by fitting the mean myocardial signal to a three‐parameter exponential curve, and applying the small flip angle approximation to the Look‐Locker correction factor 12.

The despiking algorithm was also applied to 10 artifact‐free datasets to confirm that the algorithm did not alter quantitative T_1_ estimation, by comparison of myocardial T_1_ before and after application of the despiking algorithm.

Furthermore, to assess the recovery of accurate T_1_ estimation, simulated RF noise corruption was added to an artifact‐free T_1_ dataset, and the despiking procedure was applied. To simulate RF noise, a random number and distribution of high intensity spikes were added to an artifact‐free dataset, with spike intensity rise/decay in the readout direction (to mimic spike duration in experimental observation). The same artifact‐free dataset was used as a starting point for 10 synthesized datasets. The myocardial T_1_ and the noise standard deviation were compared between the original data, synthesized spike‐corrupted data and despiked data. Noise standard deviation was used as a metric of artifact‐level, because the stripes create an increased variation in the background.

### Mouse Heart Cine Images

Mouse heart cine data was also acquired with a malfunctioning volume resonator coil (TE/TR = 1.2/4.5–5 ms, flip angle = 15°, cine frames = 20, matrix size = 128 × 128, resolution = 200 μm, slice thickness = 1 mm). Cine data was used to assess the effectiveness of the despiking algorithm for cardiac images obtained using a different acquisition scheme: the cine data differed from T_1_ mapping data because the images had greater blood–myocardium contrast, and thus a different k‐space distribution, dynamic variation in anatomy, and less frame‐to‐frame variation in overall signal intensity. This dataset was also used to demonstrate the application of the algorithm to a single static frame.

### Kidney Diffusion Tensor Imaging

DTI is especially prone to RF spiking due to its gradient‐heavy acquisition. In addition, because the signal‐to‐noise ratio is typically low, artifacts are more prominent. The despiking algorithm was applied to clinical kidney DTI data acquired on a healthy volunteer with a malfunctioning body coil generating RF spikes. DTI data of one kidney was acquired on a 3T Philips Achieva TX system (Philips Healthcare, Best, the Netherlands) using a navigator‐triggered free‐breathing spin echo EPI diffusion sequence 13 (TE/TR = 106/5000 ms, field of view [FOV] = 120 × 72 mm, slice thickness = 5.5 mm, 4 slices, 3 signal averages, matrix = 112 × 107, 15 diffusion encoding directions with b = 450 s/mm^2^ and one b = 0 s/mm^2^ reference volume).

The algorithm was separately applied to reference and diffusion‐weighted volumes, because of the large signal intensity differences between the two. Fractional anisotropy (FA) maps were calculated in MATLAB from the original RF spike‐corrupted images and the despiked corrected images, and the results were compared. Image registration was performed with the FMRIB Software Library 14 (http://fsl.fmrib.ox.ac.uk/fsl/fslwiki/FLIRT) to compensate for respiratory motion during the acquisition. To avoid registration differences between the FA maps, the same deformation parameters from the despiked corrected images were also applied to the original spike‐corrupted data.

### Brain Diffusion Tensor Imaging

To test the application of the algorithm to brain DTI, which is likely to be a common application of the despiking algorithm, images were acquired on a normal volunteer under conditions established to cause RF spikes by intermittent addition of RF noise in the scanner room. Both artifact‐free and spike‐corrupted datasets were acquired with identical parameters during the same session on a 3T scanner (MAGNETOM Trio, Siemens Healthcare, Erlangen, Germany) with a 32‐channel receiver array head coil. Spin echo EPI DTI acquisition parameters were as follows: TE/TR = 90/8400 ms, 60 slices, FOV = 220 mm, matrix = 96 × 96, BW = 2003 Hz/Px, 7 reference images (b = 100 s/mm^2^) and 61 diffusion‐weighted images (DWIs) (b = 1000 s/mm^2^) with diffusion‐weighting directions evenly distributed over the surface of a sphere 15.

The despiking algorithm was applied to both artifact‐free and spike‐corrupted datasets and the images were compared. Camino 16 was used to calculate mean diffusivity (MD) and FA. To assess the effectiveness of the despiking process, MD and FA were compared between three DTI datasets from the same volunteer: (i) the artifact‐free data, (ii) the RF spike‐corrupted data and (iii) corrected data using the despiking algorithm.

All volunteer studies were conducted with local ethics committee approval and with written informed consent provided by the subjects.

## RESULTS

Following the RPCA decomposition, the low‐rank matrix (**L**) retained the full rank of the corrupted k‐space (**M**), equal to the number of frames, in all applications. The optimal value of κ, determined visually, was found to be different for each of the four acquisition methods investigated.

### Mouse Heart T_1_ Mapping

**Figure 1:**
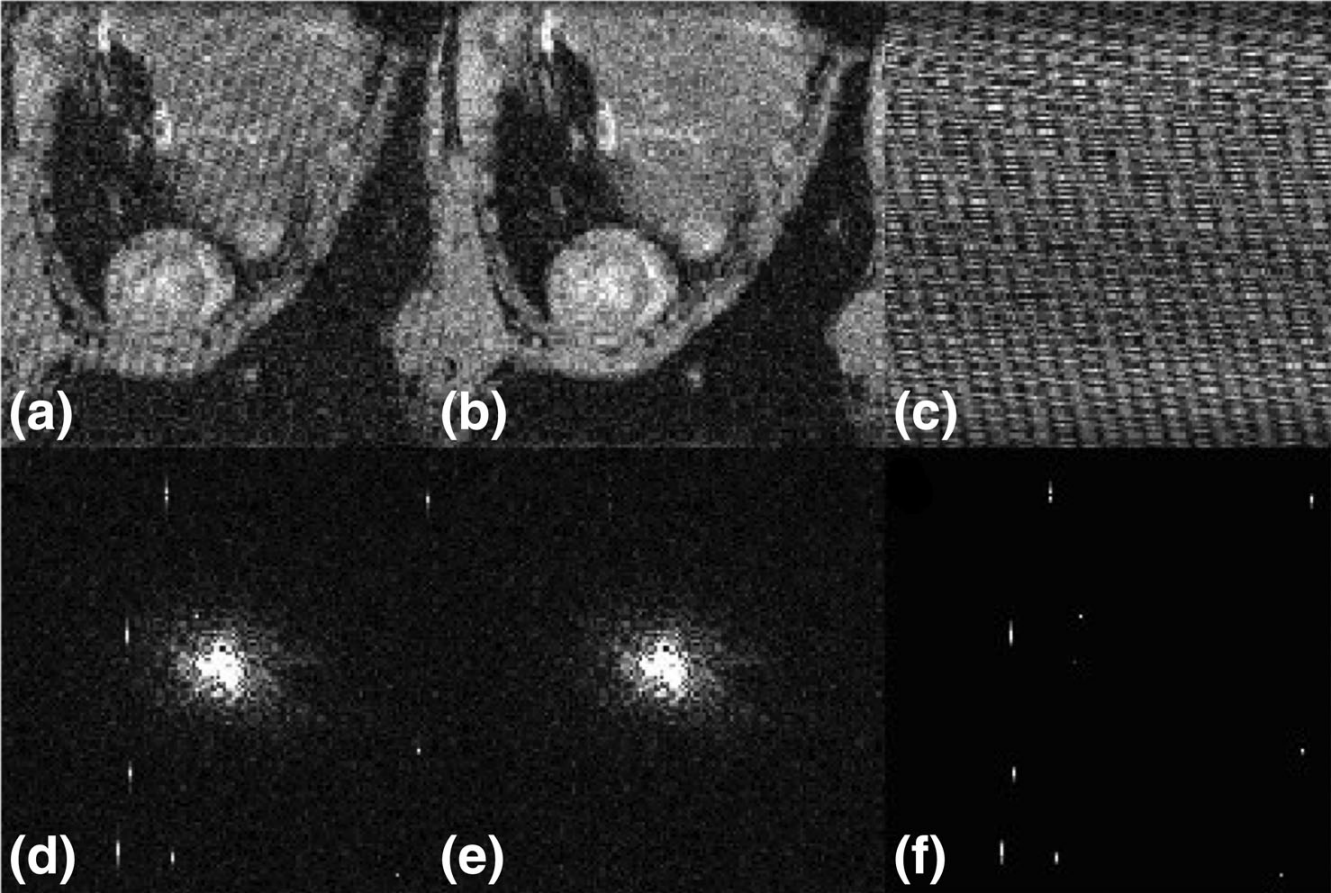
Images (a–c) and k‐space data (d–f) for a single frame of a T_1_ mapping dataset. Corrupted data (**a,d**) is decomposed into low‐rank (**b,e**) and sparse (**c,f**) components to isolate RF spikes (visible in f) and remove stripe artifact (visible in a). All the frames of the T_1_ recovery curve are shown in Supporting Video S1.

**Figure 2:**
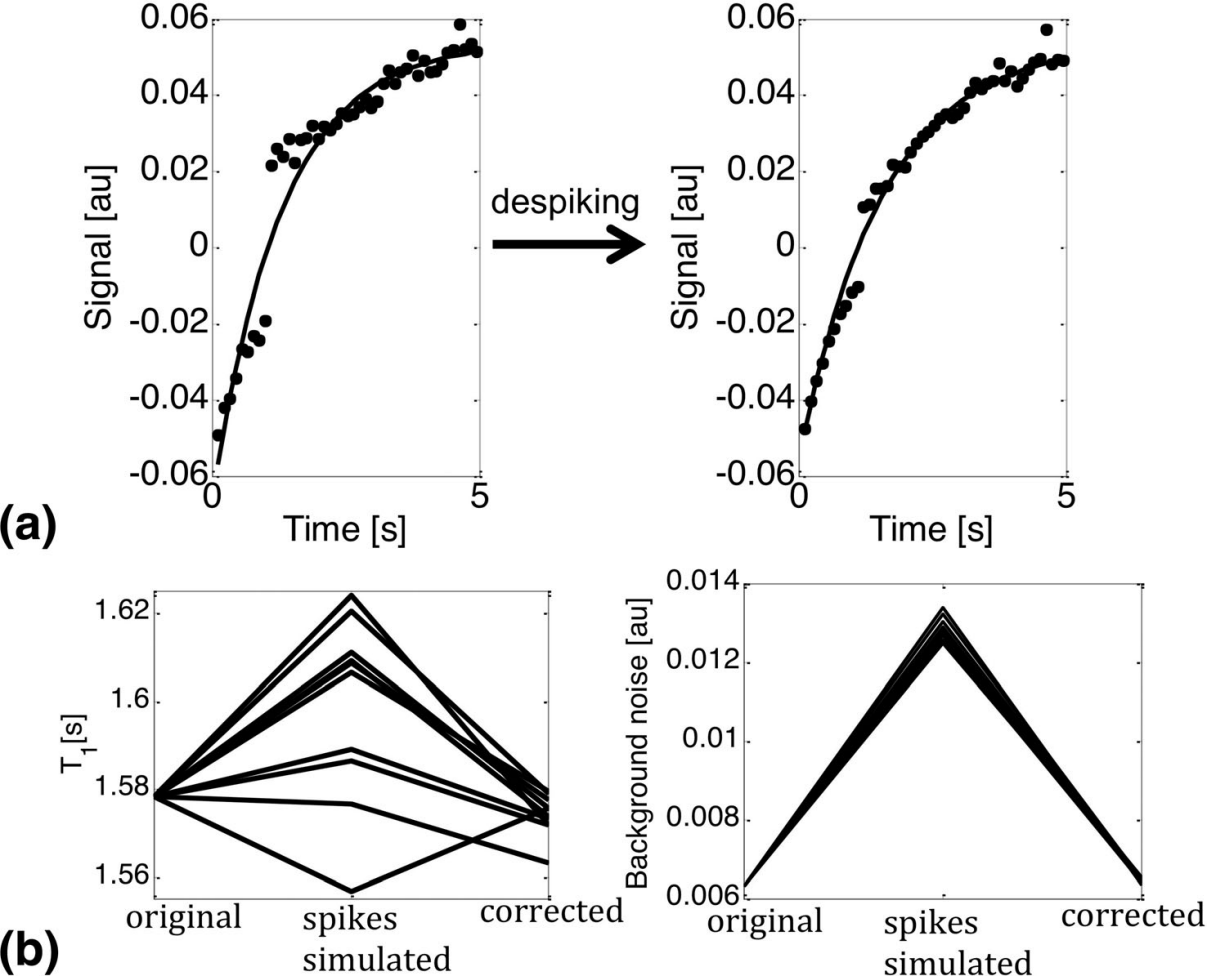
**a**: Myocardial T_1_ recovery curves from spike‐corrupted T_1_ mapping data before and after the application of the despiking algorithm. **b**: T_1_ values and standard deviation of background signal calculated from original artifact‐free images, images with added simulated RF spikes and corrected images following despiking.

The optimal κ was 5 for the mouse heart T_1_ mapping data. A clear improvement in image quality was observed following the despiking algorithm applied to spike‐corrupted data acquired using a malfunctioning RF coil (Figure 1, Supporting Video S1, which is available online). Applying the despiking algorithm to 12 spike‐corrupted datasets, myocardial T_1_ values changed by ΔT_1_ = 86.1 ± 171 ms (relative change = 4.9 ± 8.8%; *P* < 0.05 paired t‐test). The T_1_ can either increase or decrease with despiking, depending on the nature of the spikes. Figure 2a displays the T_1_ curves before and after despiking, demonstrating a reduced variability in the T_1_ recovery curve and the reduced noise floor. Remaining signal oscillation is caused by respiratory motion.

In comparison, applying the despiking algorithm to 10 artifact‐free datasets generated negligible and nonsignificant changes in myocardial T_1_ (absolute change ΔT_1_= 2.6 ± 3.0 ms, relative change = 0.16 ± 0.17%; *P* = 0.11 paired t‐test). The negligible change in T_1_ in artifact‐free data indicates that quantitative values are maintained following despiking.

Adding simulated RF spikes (depicted in Supporting Video S2) to the datasets changed T_1_ values compared with original values by 25.3 ± 14.6 ms, and applying the despiking procedure regained T_1_ values to within 4.5 ± 4.3 ms of original values (Fig. 2b). Similarly, the standard deviation of the background noise was increased by 103 ± 45% in the synthesized spike noise data, and despiking returned this to within 21.8 ± 9.4% of the original value. These results indicate that the despiking algorithm can successfully regain quantitative T_1_ values from corrupted datasets.

### Mouse Heart Cine Images

**Figure 3:**
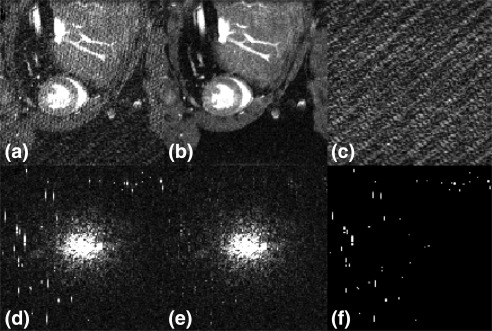
Images (a–c) and k‐space data (d–f) for a single frame of the cine dataset. Corrupted data (**a,d**) is decomposed into low‐rank (**b,e**) and sparse (**c,f**) components to isolate RF spikes (visible in f) and remove stripe artifact (visible in a). All cine frames are shown in Supporting Video S3.

The optimal κ was 6 for the mouse heart cine images. Figure 3 and Supporting Video S3 show the improvement in cine images following application of the despiking algorithm. In this case, some low intensity spike signal can still be observed, but the spike signal has been sufficiently suppressed to remove detrimental image artifacts.

**Figure 4:**
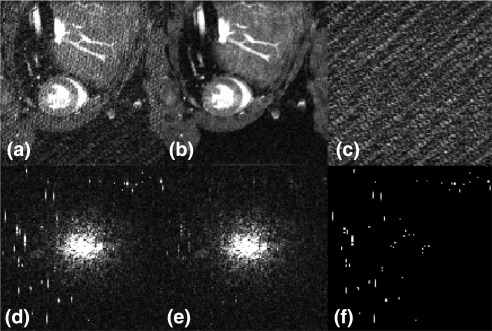
Demonstration of RPCA despiking applied to a static image. Images (a–c) and k‐space data (d–f) for a single frame from the cine dataset (frame also displayed in Figure 3). Corrupted data (**a,d**) is successfully decomposed into low‐rank (**b,e**) and sparse (**c,f**) components to isolate RF spikes (visible in f) and remove stripe artifact (visible in a).

Furthermore, the mouse heart cine data was used to demonstrate the application of this algorithm to a single frame or static image (Fig. 4). The broad central k‐space peak in the cine images makes this a challenging test of the single frame application of the RPCA despiking algorithm. The optimal κ was 3 for the static image. In this case, the RPCA algorithm performs well and isolates RF‐spikes similarly to the multi‐frame cine dataset. However, much of the peaked central cluster is initially incorrectly placed in the sparse matrix, which is then corrected in the center refilling step of the algorithm. Specifically, for the cardiac cine dataset with 20 frames, an average of 70 pixels of the central region (of the possible 256 pixels) were misclassified as sparse. In comparison, applying the algorithm to a static frame from this dataset, 161 pixels of the central region were misclassified.

### Kidney Diffusion Tensor Imaging

**Figure 5:**
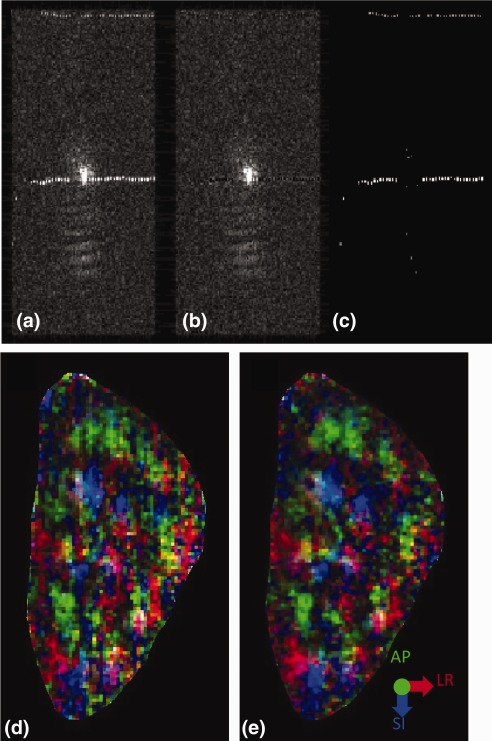
Repetitive spike pattern caused by gradient vibration observed in k‐space data (a) from kidney DTI dataset. Corrupted data (**a**) is decomposed into low‐rank (**b**) and sparse (**c**) to isolate k‐space spikes. Color FA maps from the original spike‐corrupted data (**d**) contain stipe artifacts which are removed following despiking (**e**).

For the kidney DTI dataset, the optimal κ was 5 for diffusion‐weighted images (b = 450 s/mm^2^) and 6 for reference images (b = 0 s/mm^2^), due to the difference in the signal intensity and number of frames between the reference and diffusion weighted image volumes. The spikes in the kidney DTI data had a repeated structure (Figs. 5a,c) corresponding to gradient‐induced vibrations consistent between phase encoding lines, which appeared similarly in all frames of the dataset. The FA maps showed noticeable stripe artifacts in the original spike‐corrupted image (Fig. 5d), which were removed following despiking (Fig. 5e). Furthermore, the mean FA values from the original spike‐corrupted images (cortex mean FA = 0.28; medulla mean FA = 0.38) were decreased following despiking (cortex mean FA = 0.25; medulla mean FA = 0.37). This reduction in FA following despiking suggests that the values from this volunteer are moving toward the mean values reported in a cohort of 12 healthy volunteers 13 (cortex mean FA = 0.20 ± 0.014 and medulla mean FA = 0.35 ± 0.032; mean ± standard deviation). Residual elevated FA in the cortex may be caused by incomplete spike removal at the center of k‐space following the center refilling step.

### Brain Diffusion Tensor Imaging

**Figure 6:**
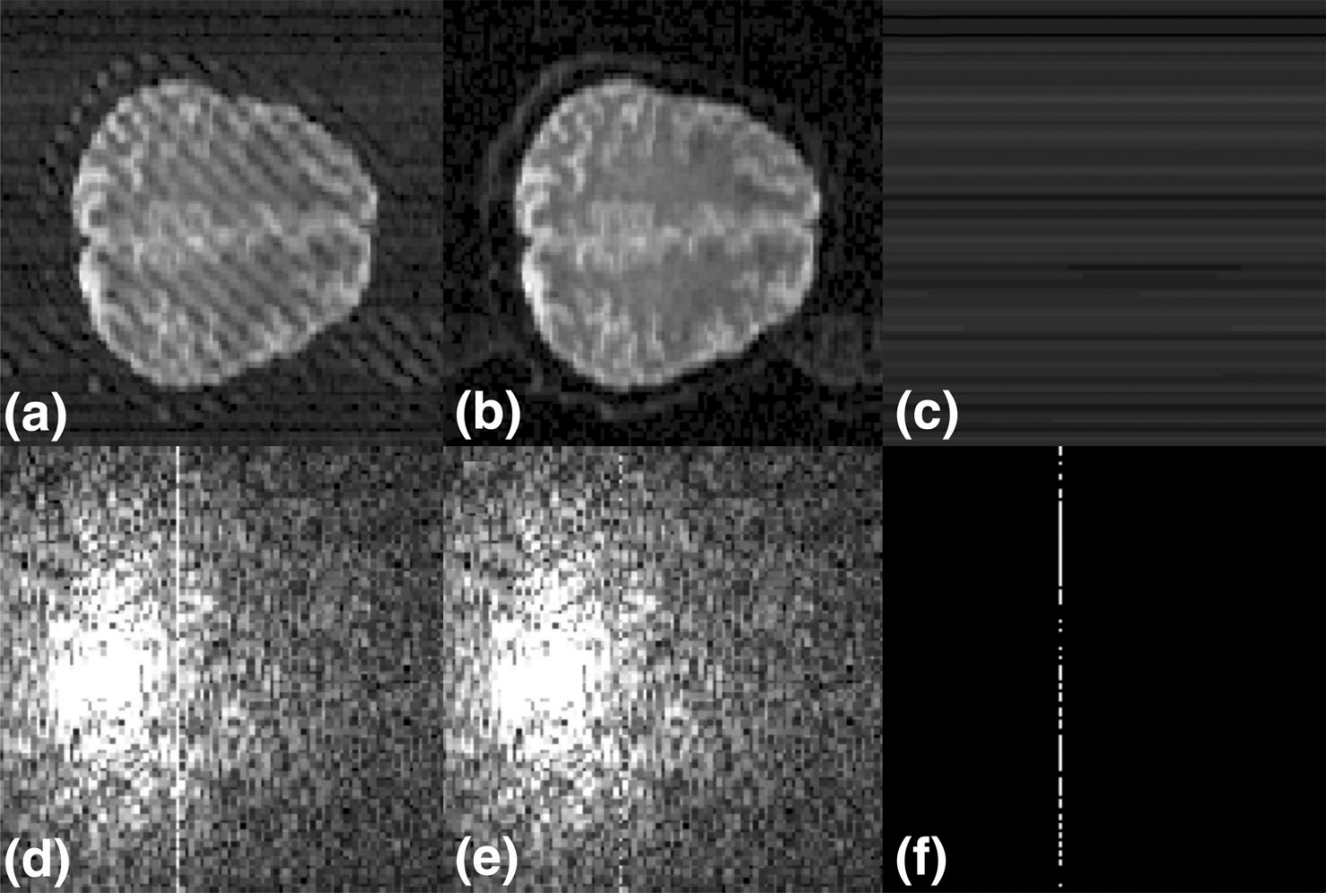
Images (a–c) and k‐space (d–f) for a single frame of the brain DTI dataset. Corrupted data (**a,d**) is decomposed into low‐rank (**b,e**) and sparse (**c,f**) components to isolate RF spikes (visible in f) and remove stripe artifact. All reference images and DWIs frames are shown from a representative slice in Supporting Video S4. The k‐space center is offset because the DTI acquisition used partial Fourier encoding in the phase‐encode direction.

For the brain DTI dataset, the optimal κ was 5 for diffusion‐weighted images (b = 1000 s/mm^2^) and 9 for reference images (b = 100 s/mm^2^), due to the difference in the signal intensity and number of frames. The despiking algorithm improved the image quality of DTI data acquired under RF spiking conditions (Figure 6, Supporting Video S4). Due to the method used to introduce RF noise in the scanner room for these measurement, the spikes in this dataset appears as a succession of high intensity data points along individual EPI readout lines. Despite this variation in the spike appearance, the RPCA despiking algorithm was still able to remove the anomalous signal. In some frames, spike intensity is lowered but not completely eliminated, but the suppression of the spike signal is sufficient to remove detrimental artifacts.

**Figure 7:**
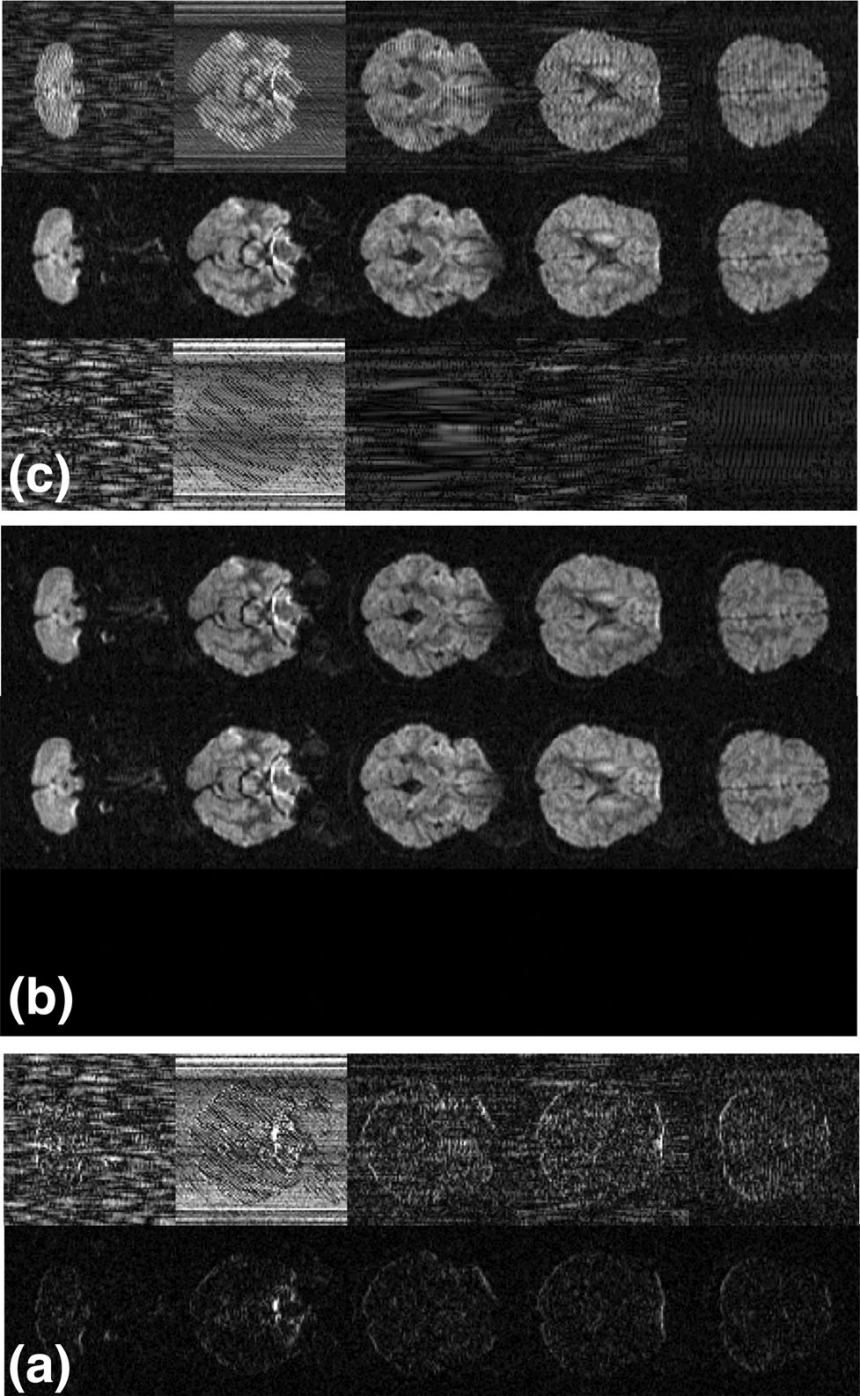
Original images (top row), despiked images (second row) and the difference between the images (bottom row) for both the spike corrupted (**a**) and the artifact‐free (**b**) brain DTI datasets. No difference between images before and after despiking is observed for the artifact‐free dataset. **c**: The difference between spike corrupted images and artifact‐free images before (top row) and after (bottom row) despiking.

**Figure 8:**
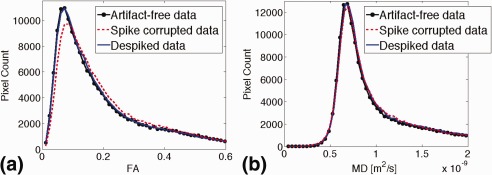
Histograms of FA (**a**) and MD (**b**) for artifact‐free data, spike‐corrupted data and corrected data using the despiking algorithm. The FA and MD values from the despiked data were equivalent to those derived from the artifact free‐data.

Figure 7 depicts images from five selected slices, showing that the despiking algorithm successfully removed artifacts from the spike‐corrupted dataset (Fig. 7a), but did not affect the images in the artifact‐free dataset (Fig. 7b). In addition, we show that despiked images matched the artifact‐free acquisition (Fig. 7c). Small differences observed between artifact‐free images and despiked images are likely explained by subtle movement and physiology changes between the two separate acquisitions. In addition, quantitative MD and FA values were equivalent in the despiked dataset to those measured from the artifact‐free dataset (Fig. 8).

## DISCUSSION

We have demonstrated that Robust Principal Component Analysis can successfully remove RF spike noise and thus reduce detrimental stripe artifacts in MRI images in a postprocessing algorithmic step. We have applied this new despiking algorithm to four different types of datasets to demonstrate the flexibility of the proposed method in k‐space spike removal. Importantly, using T_1_ mapping and DTI data, we demonstrated that quantitative parameter values are maintained in artifact free datasets and recovered from spike‐corrupted datasets when applying this algorithm.

A postprocessing algorithm to remove artifacts is particularly beneficial, because it eliminates the need to reacquire data, which can be very inefficient when performing long scans, or even impossible in the clinical setting. Furthermore, in the case where hardware requires repair or spikes are not noticed until after the scanning session, a postprocessing algorithm avoids the need to have patients return at a later date to reacquire data, or for research studies, to scan more patients or animals to replace those whose data is rendered unusable due to the presence of artifacts. Because this algorithm does not affect spike‐free data, it could even be included as a standard postprocessing step for sequences that are prone to spiking, to automatically remove any RF spikes present. However, we would like to emphasize that for optimal data quality it remains important for hardware problems to be rectified promptly, and thus, if this RPCA despiking algorithm is used as a standard processing step, it should also act as a detector for the onset of RF spiking problems, so that the scanner support engineers can be informed at the earliest opportunity.

This algorithm has advantages over previously presented methods, because it is broadly applicable to a range of data types. In comparison to threshold‐based or outlier‐based methods for spike‐detection, our RPCA‐based method does not require the data to conform to a specific template. Here, we have demonstrated the utility of the algorithm using four example data types as proof‐of‐concept. Spikes were successfully removed for all datasets of each type, providing evidence of the broad applicability of this algorithm to range of spike intensities and distributions. The nature of RF‐spike artifacts is that their appearance is unpredictable. Future work will address the application of this algorithm to naturally occurring RF spike corruption in a large cohort of brain DTI data.

The four example data types described here produced very different k‐space distributions and frame‐to‐frame signal variation. The inversion recovery T_1_ mapping data has a large dynamic range of signal intensity, because it passes through a signal null, but the change in signal is smoothly varying. In comparison, cine images have more consistent frame‐to‐frame signal intensity, but with dynamic structural changes and higher blood‐myocardium contrast in the images. In DTI, the image signal intensity varies between diffusion directions (depending on the amount of diffusion anisotropy) and from slice to slice. In addition, the k‐space center also moves slightly from frame‐to‐frame, due to eddy current effects of the diffusion gradients 17, 18. The kidney DTI data generated a test of the despiking algorithm where the RF spikes maintained a repeated predictable pattern throughout the acquisition (caused by gradient‐induced vibrations). Finally, the application to a static image from the cine data tested the despiking algorithm applied to a k_x_‐k_y_ matrix for cases of single frame imaging. Application of the despiking algorithm to these multiple different data types demonstrates the versatility of the method. RF spikes were generated from a malfunctioning RF coil (T_1_ mapping, cine, and kidney DTI), simulation (T_1_ mapping) and addition of RF noise to the scanner room (DTI). The addition of RF noise to the scanner room produced spikes with longer time constants than those caused by hardware problems, however the despiking algorithm performed equally well in reducing these more prevalent RF spikes.

The optimal multiplication factor of the default λ value (κ), used to increase the sparsity penalty, was determined to be different for each application. The value was chosen by visual inspection of **L** and **S** to successfully isolate k‐space spikes into the sparse matrix to suppress image artifacts, but not misclassify an excessive number of pixels from the center of k‐space into the sparse matrix. This is the only nonautomated step in the algorithm and future studies could investigate an automated method to select this parameter based on the number of frames and knowledge of the k‐space signal distribution.

Center refilling was applied for the 16 × 16 central pixels, to maintain image contrast. However, this step can also be problematic when spikes are contained within the central region and are refilled into the low‐rank matrix. Spikes at the center of k‐space are responsible for low spatial frequency stripe artifacts, thus the artifacts remaining following despiking are most commonly the low frequency stripes. In the DTI dataset, a mean of 2 pixels per frame were initially incorrectly placed in the sparse matrix in this central region (of the possible 256 pixels). In comparison, for T_1_ mapping dataset a mean of 63 pixels in this central region were included in the sparse matrix. This difference is a result of the different number of frames in the two datasets (50 frames for T_1_ mapping versus 420 frames for DTI reference images versus 3660 frames for diffusion‐weighted images). With more frames, the RPCA algorithm has improved ability to detect the commonality of the peak at the center of k‐space between frames and thus correctly identify this as part of the low‐rank matrix. Furthermore, for a single frame, 161 central pixels were incorrectly placed in the space matrix based on the line‐to‐line data not explained by the low‐rank component.

It is indeed expected that spike artifacts affect FA more than MD (Fig. 8). The MD can be considered an average over the DWIs. In other words, in any given voxel if a spike artifact creates an outlier, the effect in the final MD value will be very small. Even if several artifacts happen to involve the same voxel, whether the artifact results in increased or decreased signal can be considered random and will tend to cancel out in the long run. In the case of FA, even a single outlier can alter the final results because FA is calculated as a variance measure of the eigenvectors. In particular, any small perturbation from an isotropic diffusion profile (i.e., when the 3 eigenvalues are nearly identical) would be noticeable. Note how in the histogram of Figure 8a the count of low FA values decreases when spike artifact corrupts the original data. Therefore, FA can be considered to be recovered after the RPCA correction method while the stability of the results pertaining to MD in Figure 8b support the notion that the despiking algorithm will correct the artifact (in this case, the FA) while it leaves the correct signal (in this case MD) unperturbed.

This algorithm is limited by the requirement of complex k‐space data, which is not always saved in clinical studies. Thus, MRI operators must be vigilant to identify corrupted datasets and save the raw data for postprocessing, if this is not automatically done by default. Future studies will investigate the application of this algorithm to magnitude images, more commonly saved, by Fourier transforming to produce a pseudo‐k‐space 1. In the pseudo‐k‐space, the spikes produce a strong component at k=0, making it more difficult to differentiate the genuine central peak from RF spike contributions with good sensitivity. In general, multi‐frame datasets are preferred for this decomposition, because the algorithm can accurately identify the center of k‐space as consistent between frames and not misclassify it as an RF spike. Similarly, the power of the decomposition is somewhat reduced if the spikes have consistent structure between frames. Many common clinical and research imaging methods rely on a large number of images (e.g., functional MRI, DTI, arterial spin labeling, cardiac function imaging), and the presented RPCA method for correcting k‐space spike artifacts is expected to have widespread use.

In conclusion, we have presented a semi‐automated algorithm using Robust Principal Component Analysis to decompose RF spike corrupted k‐space data into sparse (RF‐spikes) and low‐rank (artifact‐free data) components. This algorithm was demonstrated on mouse heart T_1_ mapping data, mouse heart cine data and human kidney and brain DTI data to eliminate stripe artifacts and to regain quantitative parameter values derived from the images.

## Supporting information


**Video S1.** Images (top row) and k‐space (bottom row) for mouse heart T_1_ mapping data, showing the entire T_1_ recovery curve. Corrupted data (left) is decomposed into low‐rank (middle) and sparse (right) data to isolate RF spikes and remove stripe artifact.

**Video S2.** Simulated RF spikes added to artifact free mouse heart T_1_ mapping data. Corrupted data (left) is decomposed into low‐rank (middle) and sparse (right) data to remove stripe artifact in images (top row) and isolate RF spikes in k‐space (bottom row).

**Video S3.** Images (top row) and k‐space (bottom row) for mouse heart cine images. Corrupted data (left) is decomposed into low‐rank (middle) and sparse (right) data to isolate RF spikes and remove stripe artifact.

**Video S4.** Images (top row) and k‐space (bottom row) for a single slice of human brain DTI data, showing all b‐vectors for reference and diffusion‐weighted images. Corrupted data (left) is decomposed into low‐rank (middle) and sparse (right) data to isolate RF spikes and remove stripe artifact.
